# Valine 738 and lysine 735 in the fifth transmembrane domain of rTas1r3 mediate insensitivity towards lactisole of the rat sweet taste receptor

**DOI:** 10.1186/1471-2202-6-22

**Published:** 2005-04-07

**Authors:** Marcel Winnig, Bernd Bufe, Wolfgang Meyerhof

**Affiliations:** 1 German Institute of Human Nutrition Potsdam-Rehbruecke, Department of Molecular Genetics, Arthur-Scheunert-Allee 114-116, 14558 Nuthetal, Germany

## Abstract

**Background:**

The sweet taste inhibitor lactisole acts on the human sweet taste receptor heteromer TAS1R2-TAS1R3 but not on its rodent counterpart. Recently, it was shown that the lactisole sensitivity of the human sweet taste receptor involves the part of TAS1R3 encompassing the seven transmembrane regions but not the huge N-terminal domain. Using mutational analysis we investigated which amino acid residues distinguish lactisole insensitive rat from sensitive human T1R3 receptors.

**Results:**

The functional analysis of specific receptor mutants in HEK293T cells revealed that the exchange of valine 738 in the fifth transmembrane domain of rTas1r3 by an alanine is sufficient to confer lactisole sensitivity to the rat sweet taste receptor. The sensitivity of this receptor mutant is ~2 fold lower than the sensitivity of the human sweet taste receptor. Additional substitution of lysine 735 by phenylalanine in rTas1r3 results in a rat sweet taste receptor that is as sensitive to lactisole as its human counterpart. The exchange of valine 738 to alanine was accompanied by a ~50% reduction in receptor efficacy. This effect was seen with all six different sweet compounds examined.

**Conclusion:**

The lactisole insensitivity of rat sweet taste receptor is caused by only two amino acids in transmembrane region five, which is critical for the interaction of lactisole with the sweet taste receptor. The observation that the mutant receptor simultaneously displays a generally reduced sensitivity towards all agonists suggests that the lactisole insensitivity of the rodent receptor might be more likely caused by the inaccessibility of the lactisole binding site rather then by its direct disruption.

## Background

Sweet and umami taste involve TAS1R receptors, which belong to the subclass 3 of the GPCR super family and are distantly related to the V2R pheromone-, metabotropic glutamate- and calcium sensing receptors [1-3]. They have a large N-terminal extracellular domain that is linked by a cysteine-rich domain to the seven transmembrane domains [2]. Heterologous expression and functional analysis showed that cells co-transfected with *Tas1R2-TAS1R3 *responded to a variety of natural and artificial sweeteners, while the combination *TAS1R1-TAS1R3 *responded to L-amino acids [3-5]. In line with observed differences in the perception of these compounds between humans and rodents [6-8], the artificial sweeteners aspartame and cyclamate, and the sweet proteins brazzein, monellin, and thaumatin only activate the human but not the rodent TAS1R2-TAS1R3 heteromer [2,3]. Further studies of mouse-human interspecies chimeras and point-mutated receptors revealed that cyclamate interacts with the part of hTAS1R3 encompassing the seven transmembrane regions, whereas aspartame interacts with the N-terminal domain of hTAS1R2 [9]. Moreover, the cysteine-rich motif in the N-terminal domain of hTAS1R3 is required for the activation of the sweet receptor by brazzein [2]. These results and modeling studies on the basis of the solved X-ray structure of the mGLUR1 ligand binding domain strongly suggest that multiple sites in both receptor subunits are involved in the activation of the sweet receptor by its ligands [9-12]. Interestingly, differences in taste perception are not limited to sweet compounds but also have been observed for 2-(4-methoxphenoxy) propanoic acid sodium salt (lactisole), which suppresses the sweet taste of various compounds in humans but not in rats [3,13-17]. Functional studies of the human and rat sweet taste receptor recently showed that lactisole inhibits the human TAS1R2-TAS1R3 heteromer but not that of rats [3]. Moreover it could be shown that that the lactisole sensitivity of the human sweet taste receptor involves the TAS1R3 subunit [9]. Thus, the inhibition of the sweet response by lactisole in humans is due to structural differences between the rat and human sweet taste receptor. Using mutational analysis we investigated the molecular basis for these differences.

## Results and discussion

**Figure 1:**
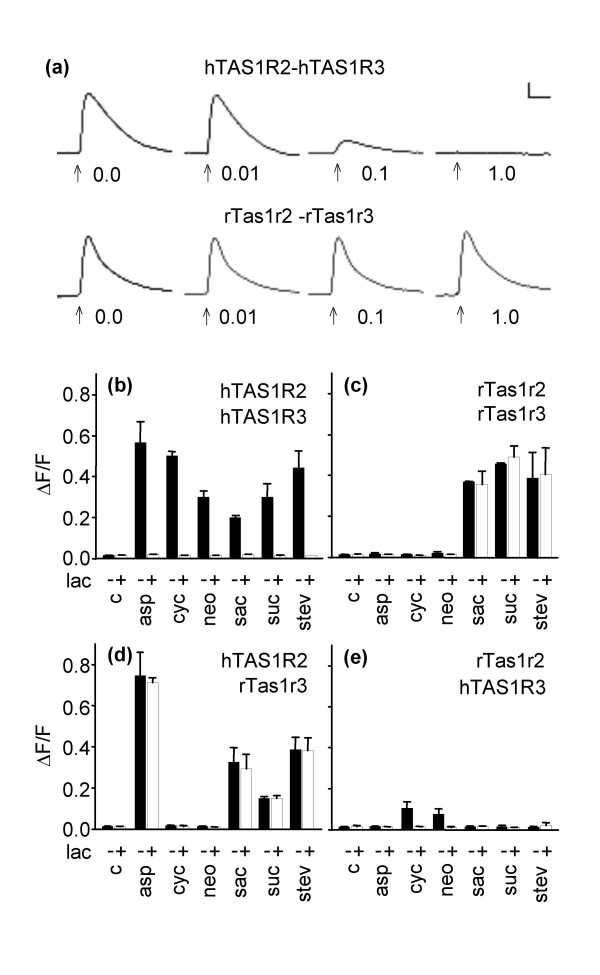
Calcium responses in cells co-transfected with different rat and human *TAS1R *subunits to stimulation with various sweeteners in the presence and absence of lactisole. (a) Intracellular calcium traces recorded in the FLIPR. Upper panel: Responses of cells cotransfected with cDNAs *hTAS1R2 and hTAS1R3*. Lower panel: Response of cells co-transfected with cDNAs *rTas1r2 and rTas1r3*. The cells were challenged with 3 mM saccharin containing 0.0, 0.01, 0.1, or 1.0 mM lactisole. Arrows denote the time of application. Signals were averaged over three independent wells of a FLIPR plate. Vertical scale, 1000 counts; horizontal scale, 1 min. (b) Cells co-transfected with *hTAS1R2-hTAS1R3*, (c) *rTas1r2-rTas1r3*, (d) *hTAS1R2-rTas1r3*, or (e) *rTas1r2-hTAS1R3*. *c*, response of mock-transfected control cells; lac, 1.0 mM lactisole; asp, 15 mM aspartame; cyc, 30 mM cyclamate; neo, 0.1 mM neohesperidine dihydrochalcone; sac, 10 mM saccharin; suc, 100 mM sucrose; stev, 1 mM stevioside; -, response without lactisole; +, response in the presence of 1.0 mM lactisole. All sweeteners were used at concentrations which were close to the maximal obtainable signal.

Application of the artificial sweetener saccharin to HEK293T-Gα_16gust44 _cells transfected with the cDNAs for the rat sweet receptor rTas1r3-rTas1r2 or its human counterpart hTAS1R3-hTAS1R2 elevated intracellular calcium levels (Fig. 1a). The responses were dose-dependent with half maximal effective concentrations (EC_50_) of 0.17 ± 0.04 mM for the human heteromer and of 1.28 ± 0.16 mM for the rat receptor heteromer. Thus, the rodent receptor is ~7 fold less sensitive to saccharin than the human receptor. This shows subtle functional differences between the human and the rat sweet receptor. In addition to saccharin also other sweet tasting compounds of various chemical classes such as the disacchride sucrose, the dipeptide derivate aspartame, the sulfamate cyclamate, the dihydrochalcone neohesperidine dihydrochalcone, and the terpenoid glycoside stevioside the activated the human receptor (Fig. 1b). Like its human counterpart, the rat sweet taste receptor could be robustly activated by saccharin, sucrose, and stevioside (Fig. 1c). But in contrast to the human receptor, aspartame, cyclamate, and neohesperidine dihydrochalcone did not activate the rat receptor (Fig. 1c) which reflects species specific variations in sweet taste perception [7,8].

Lactisole inhibited the responses of cells transfected with the cDNAs for the human receptor to all tested sweeteners (Fig. 1a, 1b) while it did not inhibit the responses of cells transfected with the rat sweet receptor (Fig. 1a, 1c). This is well in line with previous studies of the receptor function [3,9]. Based on the inhibitory effect of lactisole on the human but not on the rat receptor heteromer, we next asked which amino acids cause the functional difference of the receptors. We therefore analyzed cells co-transfected with cDNAs of the interspecies sweet taste receptor combinations, i.e. *hTAS1R3-rTas1r2 *or *rTas1r3-hTAS1R2*. The combination hTAS1R2-rTas1r3 responded to aspartame, saccharin, sucrose, and stevioside but not to cyclamate and neohesperidine dihydrochalcone (Fig. 1d). The responses induced by all four agonists were lactisole insensitive (Fig. 1d). The combination rTas1r2-hTAS1R3 clearly responded to the sweeteners cyclamate and neohesperidine dihydrochalcone, although with markedly reduced amplitudes. Aspartame, saccharin, sucrose, and stevioside failed to activate this receptor combination (Fig. 1e). This indicates that the hTAS1R3 subunit is involved in the response of the human sweet taste receptor to cyclamate and neohesperidine dihydrochalcone. Notably, the responses were lactisole sensitive (Fig. 1e). In line with previous observations [9] we therefore conclude that the lactisole sensitivity is solely mediated by the hTAS1R3 subunit.

**Figure 2:**
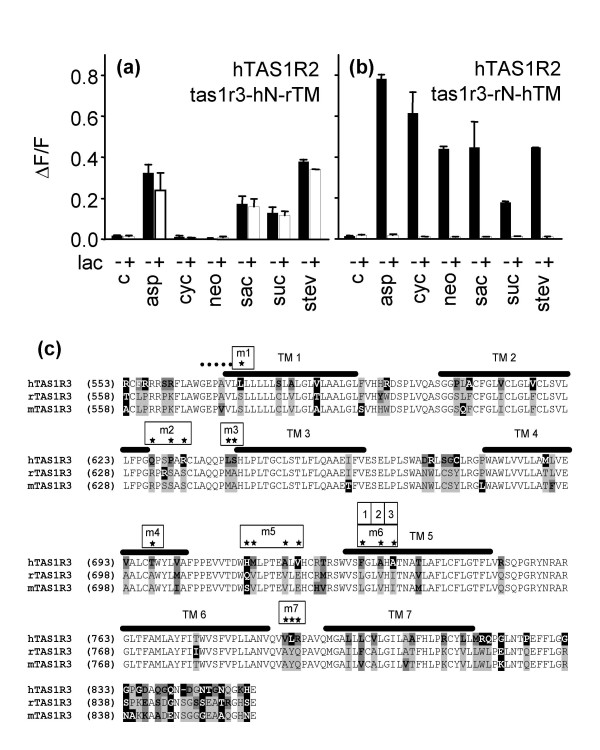
Effect of lactisole on Tas1r3 variants. (a) Calcium responses of cells co-transfected with *hTAS1R2 *and a *Tas1r3 *chimera comprising the human N-terminus and the rat transmembrane region or *hTAS1R2 *and a *Tas1r3 *chimera comprising the rat N-terminus and the human transmembrane region (b) to stimulation with various sweeteners in the presence and absence of lactisole. *c*, response of mock-transfected control cells; lac, 1.0 mM lactisole; asp, 15 mM aspartame; cyc, 30 mM cyclamate; neo, 0.1 mM neohesperidine dihydrochalcone; sac, 10 mM saccharin; suc, 100 mM sucrose; stev, 1 mM stevioside; -, response without lactisole; +, response in the presence of 1.0 mM lactisole. All sweeteners were used at concentrations which were close to the maximal obtainable signal. (c) Alignment of amino acid sequences of the human, rat, and mouse Tas1r3 transmembrane regions. Color code: white, identical; grey, conservative; dark-grey, similar; black, non-similar. TM, predicted transmembrane domain, indicated by a black bar. Dots indicate the location of the primer used to generate the chimeric rat-human Tas1r3 receptors. Asterisks denote amino acid positions that are identical in rat and mouse but variable between humans and rodents and are located in the extracellular loops or the upper part of the transmembran regions. Boxes indicate the several rTas1r3 mutants m1 – m7, including the amino acid substitutions. m6/1 – m6/3 signified the reconverted amino acid of mutant m6.

Next we wanted to know which part of hTAS1R3 mediates the sensitivity to lactisole. To address this point we constructed two chimeric receptors. In the receptor chimera tas1r3-rN-hTM we fused the N-terminal domain of rTas1r3 to the C-terminal part containing the transmembrane regions of hTAS1R3. In the complementary chimera tas1r3-hN-rTM the human N-terminal domain was fused to the segment of the rat subunit encompassing the transmembrane regions. Cells co-transfected with plasmids encoding hTAS1R2 and tas1r3-hN-rTM responded to aspartame, saccharin, sucrose, and stevioside but not to cyclamate and neohesperidine dihydrochalcone (Fig. 2a). The responses to all four agonists were lactisole insensitive (Fig. 2a). Cells co-transfected with plasmids encoding hTAS1R2 and tas1r3-rN-hTM responded to all sweeteners tested (Fig. 2b). The responses to all six agonists were lactisole sensitive (Fig. 2b). Based on these findings we conclude that the activation the human sweet receptor by cyclamate and neohesperidine dihydrochalcone involves the hTAS1R3 transmembrane regions. Moreover, the sensitivity towards lactisole is determined by the hTAS1R3 transmembrane regions.

**Figure 3:**
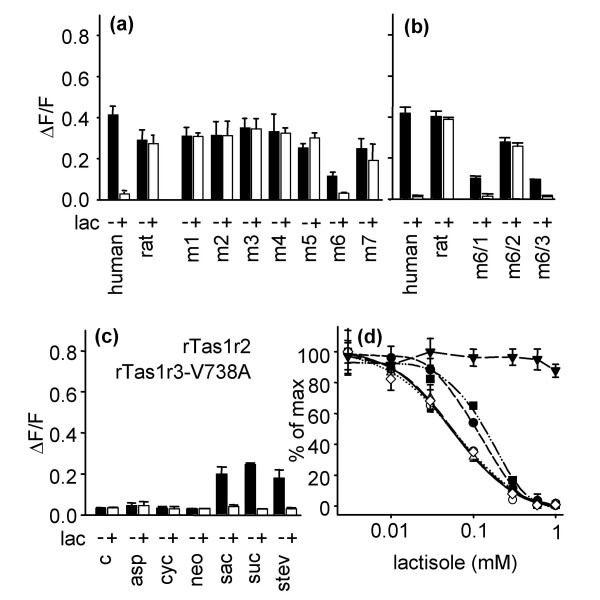
Effect of lactisole on Tas1r3 variants. (a, b) Responses of cells co-transfected with *rTas1r2 *and *rTas1r3 *mutants to stimulation with 1 mM stevioside. (c) Calcium responses of cells co-transfected with *rTas1r2 *and the *rTas1r3-*V738A receptor variant to stimulation with various sweeteners in the presence and absence of lactisole. *c*, response of mock-transfected control cells; lac, 1.0 mM lactisole; asp, 15 mM aspartame; cyc, 30 mM cyclamate; neo, 0.1 mM neohesperidine dihydrochalcone; sac, 10 mM saccharin; suc, 100 mM sucrose; stev, 1 mM stevioside; -, response without lactisole; +, response in the presence of 1.0 mM lactisole. All sweeteners were used at concentrations which were close to the maximal obtainable signal. (d) Concentration-dependent inhibition of calcium responses by lactisole in cells transfected with *hTAS1R2-hTAS1R3 *(open circles, solid line), *rTas1r2-rTas1r3 V738A *(squares, dash-dot-dotted line), *rTas1r2-rTas1r3 m6 *(open triangles up, long dashed line), *rTas1r2-rTas1r3 m6/1 *(filled circles, short dashed line), *rTas1r2-rTas1r3 m6/2 *(triangles down, medium dashed line) or *rTas1r2-rTas1r3 m6/3 *(open diamond, dotted line) that were challenged with 3 mM stevioside.

Sequence comparisons (Fig. 2c) between hTAS1R3 and its rat and mouse orthologs revealed 75 amino acid differences in the transmembrane regions. As mice and rats are lactisole insensitive while humans are lactisole sensitive, we reasoned that the amino acids responsible for this species difference are conserved in the rodent receptors but variable between rodents and humans. We therefore excluded 27 of the 75 amino acids because they vary between rat and mouse. Due to the polar nature of lactisole we assumed that it is membrane impermeable and should therefore interact with amino acids that are accessible from the extracellular site. Based on this assumption we identified 17 amino acids (Fig. 2c, asterisks) as the most promising candidates. We assumed that the conversion of one or several of these amino acids from the rodent to the human form should confer the lactisole sensitivity to the rat receptor. To test this hypothesis we created seven rTas1r3 variants (m1 – m7) that contained 1–4 amino acids of hTAS1R3 (Fig. 2c, boxes). The functional analysis of these receptor variants revealed that receptor mutant m6 confered lactisole sensitivity to the heteromer, while all other *rTas1r3 *mutants did not (Fig. 3a). Thus, the 3 amino acids exchanges L735F, V738A, and I740A of mutant m6 are sufficient to confer lactisole sensitivity to rTas1r3. To test whether all exchanges in m6 are equally important, we created three variants, m6/1, m6/2 and m6/3 (Fig. 2c, boxes). In each of these, we reconverted one of the three human amino acids present in the mutant m6 back into that of the rat receptor. In case the amino acid is important for the inhibition of the sweet receptor, the variant should loose its lactisole sensitivity. Only mutant m6/2, L735F and I740A, was lactisole insensitive, while the two other variants m6/1, V738A and I740A, and m6/3, L735F and V738A remained lactisole sensitive (Fig. 3b). Thus, we concluded that the alanine to valine exchange in position 738 in transmembrane segment 5 is crucial for the lactisole sensitivity. To confirm this, we created a rTas1r3 variant where we only substituted the valine at amino acid position 738 by an alanine. Functional analysis of this receptor variant, rTas1r3-V738A, co-transfected with rTas1r2 revealed that it was activated by saccharin, sucrose, and stevioside, but not by aspartame, cyclamate and neohesperidine dihydrochalcone (Fig. 3c). Thus, rTas1r3-V738A shows the same agonist profile as the wild type rat receptor (Fig. 3c, 1b). However, in marked contrast to the rat wild type receptor, the response of rTas1r3-V738A was always lactisole sensitive (Fig. 3c).

To test if rTas1r3-V738A displays the same degree of lactisole sensitivity as the human receptor, we determined the half maximal inhibitory concentration (IC_50_) of lactisole on rTas1r3-V738A and compared it with the IC_50 _values of the human receptor and the mutants m6, m6/1, m6/2, and m6/3. Although all variants except the lactisole insensitive mutant m6/2 were inhibited by lactisole, we observed subtle differences (Fig. 3d). The IC_50 _values were 0.05 ± 0.01 for the human receptor, 0.06 ± 0.01 for m6, 0.11 ± 0.01 m6/1, 0.05 ± 0.02 for m6/3 and 0.16 ± 0.03 for rTas1r3-V738A (Fig. 3d). Thus, the human receptor and the mutants m6 and m6/3 behave identical while the mutants m6/1 and rTas1r3-V738A are ~2 fold less lactisole sensitive. This result shows that the replacement of lysine 735 by phenylalanine is required for full lactisole sensitivity of the mutant rat receptor.

**Figure 4:**
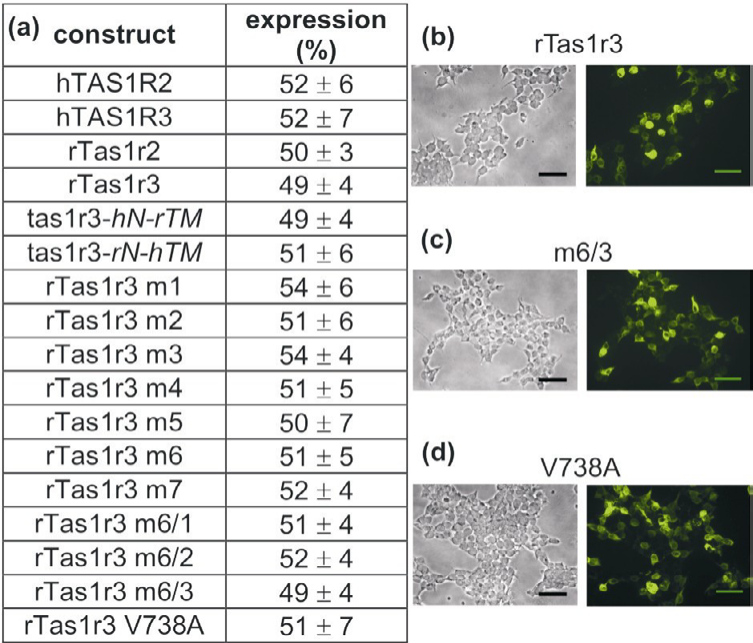
Expression of receptor variants. The table shows the overall expression in % of all receptor constructs detected by immuncytochemistry (a), transmission and fluorescence pictures of HEK293T/ G_α16gust44 _cells transfected with *rTas1r3 *(b), *rTas1r3-V738A *(c) or *rTas1r3 m6/3 *(d) DNA. Scale, 50 μm.

Notably, the gain of lactisole sensitivity in the rodentTas1r3 variants m6, m6/3, m6/1, is accompanied by a ~50% reduction of the signal amplitude compared to the wild type rTas1r3 (Fig. 3a,3b). In marked contrast, the loss of lactisole sensitivity by the rTas1r3 mutant m6/2 led to an increase in the signal amplitude (Fig. 3b). The diminished signal amplitudes are not caused by individual differences in the expression rates of the receptor variants (Fig. 4). Moreover the V738A receptor variant, that contains just a single amino acid exchange also showed a ~50 % reduced amplitude compared to the native rat sweet taste receptor (Fig. 3b, 3c). This reduction is independent of the sweet taste agonist (Fig. 3c). The activation of the TAS1R2-TAS1R3 receptor by different sweeteners involves several receptor sites [9-12]. As the V738A amino acid exchange leads to an agonist independent amplitude reduction, this observation in combination with its localization in the fifth transmembrane region argues for a global or regional alteration of the receptor structure instead of a specific change at the agonist binding sites.

## Conclusion

The lactisole insensitivity of rat sweet taste receptor is caused by valine 738 in transmembrane region five. The additional replacement of a lysine residue by a phenylalanine at amino acid position 735 again in transmembrane region five is sufficient to increases the lactisole sensitivity of the mutant rat receptors to the level of the human counterpart. Thus, transmembrane region five is critical for the interaction of lactisole with the sweet receptor. The observation that the mutant receptors displays a generally reduced sensitivity towards all agonists suggests that the lactisole insensitivity of the rodent receptor might be more likely caused by an inaccessibility of the lactisole binding site rather then by its direct disruption. Instead the lactisole insensitivity of the rodent receptor more is likely a side effect that is caused by a steric alteration in the fifth transmembrane region of the rat TAS1R3 subunit induced by the valine at amino acid 738. Recently functional studies of the mouse and human sweet taste receptor in combination with mutational analysis led to a model of the lactisole binding site in human TAS1R3 [18]. This model predicts that the alanine 733 in human TAS1R3 which corresponds to valine 738 of rat TAS1R3 is not directly involved in lactisole binding although it lies in close proximity to the lactisole binding pocket. The proximity of this amino acid to the predicted lactisole binding pocket further supports our conclusion that valine 738 in rat sweet taste receptor prevents the access of lactisole to its binding site.

## Methods

### Cloning of the TAS1R cDNAs

*hTAS1R2 *was constructed via amplification of all exons from genomic DNA by recombinant polymerase chain reactions (PCR) [19]. *hTAS1R3 *was cloned from testis cDNA. *rTas1r2 *was cloned from rat vallate papillae cDNA. PCR products were cloned into a pcDNA5/FRT expression vector (Invitrogen), fused with a C-terminal FLAG-epitope for immuncytochemical detection, sequenced, and checked for comparable protein expression by immuncytochemistry of transfected HEK293T cells.

### Construction of chimeric receptor cDNAs

The receptor chimeras of Tas1r3 were constructed using recombinant PCR. In the first reactions the N-terminal and the C-terminal part containing the seven transmembrane domains of *hTAS1R3 *and *rTas1r3 *were amplified from plasmid DNA using Ultra Pfu Polymerase (Stratagene). For the *hTAS1R3 *and *rTas1r3 *N-termini the forward vector-anchored primer (CMV: CGCAAATGGGCGGTAGGCGTG) and a reverse gene specific primer (CACAGCCGGCTCGCCC) were used. The *hTAS1R3 *and *rTas1r3 *C-terminal part were amplified with forward gene specific primer (GGGCGAGCCGGCTGTG) and a reverse vector-anchored primer (TAGAAGGCACAGTCGAGG). PCR conditions were: 5 min, 94°C, 24 cycles 1 min 94°C, 0.5 min 64°C, 2 min 72°C; 5 min 72°C. The amplicons were purified using the Highpure PCR Product Purification Kit (Roche) and used as template for the second PCR. To create the chimeric receptor tas1r3-hN-rTM, equimolar amounts (~50 ng) of the *hTAS1R3 *N-terminus and *rTas1r3 *transmembrane domains were combined without additional oligonucleotides. PCR conditions were: 5 min 94°C; 15 cycles of 2 min 64°C, 2 min 72°C and 0.5 min 94°C; followed by 5 min 64°C and 10 min 72°C. The identical protocol and combining the *rTas1r3 *N-terminus with the human transmembrane regions resulted in receptor chimera tas1r3-rN-hTM. These chimeras were cloned into pcDNA5/FRT.

### Construction of Tas1r3 mutants

Receptor mutants were generated by site directed mutagenesis according to the QuikChange protocol (Stratagene). The forward and reverse complement primers contained the desired mutations and annealed to the same sequence on opposite strands of the plasmids. The following *rTas1r3 *receptor variants were generated: m1: S576L; m2: R632Q, S635P, S637Q; m3: M644L A645S; m4: A702T; m5: Q717H, V718M, V723A, E725V; m6: L735F, V738A, I740A. m6/1: V738A, I740A, m6/2: L735F, I740A, m6/3: L735F, V738A. m7: A793V, Y794L, Q795R. Subsequently a *rTas1r3*-V738A variant was generated. All constructs were checked by sequencing.

### Functional expression

The cDNAs were transiently transfected into HEK293T cells stably expressing the chimeric G-protein subunit Gα_16gust44 _[20] using Lipofectamine 2000 (Invitrogen) according to the manufacturer's protocol. 3–4 hours after transfection, DMEM was replaced by low-glucose DMEM supplemented with GlutaMAX and 10% dialyzed FBS (Invitrogen). 22–44 hours later, cells were loaded for 1 hour with the calcium sensitive dye Fluo4-AM (2 μg/ml in DMEM, Molecular Probes). Cells were washed 3x in solution C1 (130 mM NaCl, 5 mM KCl, 10 mM Hepes, 2 mM CaCl_2, _and 5 mM Glucose, pH 7,4). Calcium mobilization was monitored by an automated fluorometric imaging plate reader (FLIPR, Molecular Devices). Ligands (Sigma-Aldrich, Merck) were dissolved in C1 solution. All data were collected from at least two independent experiments carried out in triplicate. The obtained calcium signals were corrected for the response of mock transfected cells and normalized to the fluorescence of cells prior to the stimulus using ΔF/F=(F-F0)/F0. Concentration-response curves and EC_50 _and IC_50 _values were calculated in SigmaPlot by nonlinear regression using the function f = ((a-d)/(1+(x/EC_50_)^nH^)+d) and f = (a-b)/ [1+(x/IC50)^nH^]+b respectively.

### Immuncytochemistry

HEK-293T/G_α16gust44 _cells were seeded on coverslips coated with 10 μg/ml poly-D-lysine and transfected with the respective cDNAs. 48 h after transfection cells were washed with PBS and fixed and permeabilized for 5 min in acetone:methanol (1:1). Non-specific binding was reduced by incubating the cells in 5% goat serum for 1 h. To detect the receptors, antiserum against the FLAG-epitope (anti-FLAG M2 (Sigma), 1:2000 in 3% goat serum) was added to the cells for 1 h at room temperature (RT). After washing the cells three times with PBS we added Alexa488-conjugated goat antiserum against mouse IgG ((Molecular Probes), 1:1000 in 3% goat serum) for 1 h at RT. The cells were embedded in Fluorescent Mounting Medium (Dako) and analyzed using a fluorescence microscope (Zeiss Axioplan, Jena) and a camera (RT Slide, Visitron Systems, Munich).

## List of abbreviations

GPCR G-protein coupled receptor

HEK293T/ Gα_16gust44 _Human embryonic kidney cells stably expressing the

large T antigen and the G protein chimera Gα_16gust44_

EC_50 _half maximal effective concentration

IC_50 _half maximal inhibitory concentration

FLIPR fluorometric imaging plate reader

## Authors' contributions

MW cloned most of the TAS1R subunits, constructed the chimeric TAS1R3 receptors and carried out all functional studies. BB created the receptor variants m1–m7, participated in the study design and drafted the manuscript. W.M conceived the study, participated in its design and coordination and helped to draft the manuscript. All authors read and approved the final manuscript.
